# Saving Lives at Birth; development of a retrospective theory of change, impact framework and prioritised metrics

**DOI:** 10.1186/s12992-018-0327-z

**Published:** 2018-01-29

**Authors:** Marek Lalli, Harriet Ruysen, Hannah Blencowe, Kristen Yee, Karen Clune, Mary DeSilva, Marissa Leffler, Emily Hillman, Haitham El-Noush, Jo Mulligan, Jeffrey C. Murray, Karlee Silver, Joy E. Lawn

**Affiliations:** 1 Maternal, Adolescent, Reproductive and Child Health (MARCH) Centre, Department of Infectious Disease Epidemiology, London School of Hygiene and Tropical Medicine, London, WC1E 7HT UK; 2 Grand Challenges Canada / Grands Défis Canada at the Sandra Rotman Centre, MaRS Centre, South Tower, 101 College Street, Suite 406, Toronto, ON M5G 1L7 Canada; 3 Center for Accelerating Innovation and Impact, United StatesNorwegian Agency for International Development (USAID) Cooperation, 1300 Pennsylvania Ave. NW, Washington DC, USA; 4 Department for Global Health, Education and Research, Norwegian Agency for Development Cooperation, Ruseløkkveien 26, 0251 Oslo, Norway; 5 Maternal and Child Health Division, Bureau for Global Health, United States Agency for International Development (USAID), 1300 Pennsylvania Ave., NW, Washington DC, USA; 6 Center for Accelerating Innovation and Impact, Bureau for Global Health, United States Agency for International Development (USAID), 1300 Pennsylvania Ave. NW, Washington DC, USA; 7 Health Team, Research and Evidence Division, Department for International Development, 22 Whitehall, London, SW1A 2EG UK; 8 Discovery and Translational Sciences, Bill and Melinda Gates Foundation, 5th Avenue, Seattle, WA 98119 USA

**Keywords:** Theory of change, Neonatal health, Maternal health, Stillbirth, Indicators, Innovation, Impact metrics

## Abstract

**Background:**

Grand Challenges for international health and development initiatives have received substantial funding to tackle unsolved problems; however, evidence of their effectiveness in achieving change is lacking. A theory of change may provide a useful tool to track progress towards desired outcomes. The Saving Lives at Birth partnership aims to address inequities in maternal-newborn survival through the provision of strategic investments for the development, testing and transition-to-scale of ground-breaking prevention and treatment approaches with the potential to leapfrog conventional healthcare approaches in low resource settings. We aimed to develop a theory of change and impact framework with prioritised metrics to map the initiative’s contribution towards overall goals, and to measure progress towards improved outcomes around the time of birth.

**Methods:**

A theory of change and impact framework was developed retrospectively, drawing on expertise across the partnership and stakeholders. This included a document and literature review, and wide consultation, with feedback from stakeholders at all stages. Possible indicators were reviewed from global maternal-newborn health-related partner initiatives, priority indicator lists, and project indicators from current innovators. These indicators were scored across five domains to prioritise those most relevant and feasible for Saving Lives at Birth. These results informed the identification of the prioritised metrics for the initiative.

**Results:**

The pathway to scale through Saving Lives at Birth is articulated through a theory of change and impact framework, which also highlight the roles of different actors involved in the programme.

A prioritised metrics toolkit, including ten core impact indicators and five additional process indicators, complement the theory of change. The retrospective nature of this development enabled structured reflection of the program mechanics, allowing for inclusion of learning from the first four rounds of the program to inform implementation of subsequent rounds.

**Conclusions:**

While theories of change are more traditionally developed before program implementation, retrospective development can still be a useful exercise for multi-round programs like Saving Lives at Birth, where outputs from the development can be used to strengthen subsequent rounds. However, identifying a uniform set of prioritised metrics for use across the portfolio proved more challenging. Lessons learnt from this exercise will be relevant to the development of pathways to change across other Grand Challenges and global health platforms.

**Electronic supplementary material:**

The online version of this article (10.1186/s12992-018-0327-z) contains supplementary material, which is available to authorized users.

## Background

The last century has seen unprecedented improvement in the health and development status of most populations globally; however, progress has not been equally distributed. The need to accelerate progress has been recognised, and this need intensified as we move forward in the era of the Sustainable Development Goals, seeking that ‘no-one be left behind’. Grand Challenges for international heath and development initiatives have grown from the premise that science and technology, when applied appropriately can have transformational effects, and that engaging non-traditional potential innovators and problem solvers from around the world around critical problems is key in identifying more innovations that work.

These successful innovations have the potential to be scaled-up and lead to improvements in health and development status. Millions of dollars have been invested in Grand Challenges in these areas; however, evidence of their effectiveness in achieving change is frequently lacking. A theory of change, which provides a comprehensive description of how and why a desired change is intended to happen, mapping out all the required steps in order to achieve long-term goals is a useful tool to track progress towards specific goals [1]. It includes indicators for each stage of the pathway which create a holistic impact or outcomes framework including both process and outcome indicators [2], yet these have been rarely used within the Grand Challenge context.

In this paper, we describe the process and utility of retrospectively developing a theory of change using an important Grand Challenge, Saving Lives at Birth, as a case study of the potential benefit of such a tool to other Grand Challenge programs as well as other multi-round donor programmes.

### What is the problem?

It is estimated that each year around 303,000 mothers die due to pregnancy-related causes, 2.7 million babies die within 28 days after birth and 2.6 million babies are stillborn [3–5]. Despite the existence of known and effective interventions to reduce these deaths, these options are often not available or accessible to women and newborns around the time of birth in resource constrained settings [6]. Over 40% of all maternal deaths, a third of neonatal deaths and half of all stillbirths occur during labour and on the day of birth [7]. Most of these deaths are in low- and middle-income countries (LMIC) where barriers to accessing health services for women and families are common, and high quality care is frequently hampered by a lack of human and material resources, such as electricity, clean water and adequately skilled healthcare workers [8, 9]. While the era of the Millennium Development Goals yielded great improvements in maternal and child mortality, mothers and children continue to die from preventable causes, representing an unfinished agenda which needs to be prioritised as we transition to the Sustainable Development Goals.

### What is the Saving Lives at Birth partnership?

Saving Lives at Birth: A Grand Challenge for Development aims to accelerate substantial and sustainable progress in reducing maternal and newborn deaths and stillbirths at the community level by identifying and supporting ground-breaking prevention and treatment approaches [9]. The program seeks to harness the collective imagination and ingenuity of diverse experts to develop, test, and scale innovative ideas that have the potential to leapfrog conventional approaches.

As a Grand Challenge for Development, Saving Lives at Birth is rooted in two fundamental beliefs: that innovation, when applied appropriately, can transform substantial development challenges into solvable problems; and that engaging the world in the quest for solutions is critical to instigating breakthrough progress [9, 10].

In the search for such innovative solutions, Saving Lives at Birth promotes Integrated Innovation: the coordinated application of scientific/technological, social and business innovation, in the development of solutions to complex challenges. This approach does not discount the singular benefits of each of these types of innovation alone, but rather highlights the powerful synergies that can be realised by aligning all three. Integrated Innovation recognises that scientific/technological innovation has a greater chance of going to scale and achieving global impact and sustainability if it is developed from the outset with appropriate social and business innovations. Similarly, it recognises that social or business innovations will not be effective on their own [9, 10].

### How is this being achieved?

Saving Lives at Birth is a partnership that brings together the United States Agency for International Development (USAID), Grand Challenges Canada, the Bill & Melinda Gates Foundation, the Government of Norway/Norwegian Development Agency (Norad), the United Kingdom’s Department for International Development (DFID) and, since 2015, the Korea International Cooperation Agency (KOICA) to collectively pool their resources to tackle this challenge. First launched in 2011, the partners committed to and released four annual rounds of requests for proposals, seeking innovative approaches across three domains: (1) science/technology; (2) service delivery; and (3) demand creation. In 2014, with the objective to renew the partnership for an additional four rounds, the partners sought to create a theory of change and impact framework with prioritised metrics to increase understanding of how best to position the overall program and its investments for sustained impact. This was also an opportunity to formalise the monitoring and evaluation cycles already embedded within the platform. Round 5 of Saving Lives at Birth was released in 2015. Successful applicants are awarded one of three types of funding (Table 1). Of note, the addition of the validation stage is new in Round 5, following wide consultation with stakeholders throughout this process, and the renewal of the partnership in 2014. To date, Saving Lives at Birth has awarded 108 grants to 92 unique innovations [9].

**Table 1:** Saving Lives at Birth; types of awards

		Award $US
Seed Award	To support the development and validation of ideas capable of impacting health outcomes for pregnant women and their babies in low-resource settings.	Max 250 for up to 2 years
Validation Award (new to round 5)	To introduce and validate the effectiveness of innovations to reach proof-of-concept.	Max 250 for up to 2 years
Transition-to-scale (TTS) Award	To develop, refine, and rigorously test the impact of integrated solutions that have previously measured promising health outcomes in a controlled or limited setting and have the potential to credibly scale to improve the lives of millions of pregnant women and newborns in multiple countries. Transition funding is limited to integrated solutions that unite technology, service delivery, and demand.	Max 2million for up to 4 years

### Developing a theory of change and impact framework with prioritised metrics for saving lives at birth

Here we use a theory of change and an impact framework as separate but inter-related tools, with a theory of change describing how and why an initiative works [11], or delineating a plausible and testable pathway to achieving the pre-set goals [12, 13]. Programmes can use a theory of change for planning interventions, monitoring and evaluation, engagement of stakeholders, and to describe how a process works in evidence-based policy and practice. Furthermore, developing a theory of change provides an opportunity for learning about perceived relationships in a pathway to change and can inform future directions through analysis of lessons learnt. Here, the impact framework has been designed to also capture the indicators that have been previously collected by the projects and is set to function in harmony with the theory of change. This tool aims to track the programme’s progress towards achieving its end-goals, in addition to capturing key links transcending up the theory of change.

While the Saving Lives at Birth partners had envisioned how the most successful innovations would be scaled through both public programs and private markets, a detailed theory of change had never been articulated. The Saving Lives at Birth partners therefore commissioned the development of a theory of change with the aim of reviewing the process and progress of the program to date, and to inform strategic planning for the future; with particular focus on achieving, tracking and reporting scaled impact. Since 2011, innovators have reported against a standard set of health and innovation-related metrics in addition to their project specific monitoring. While these were being aggregated across the portfolio, the metrics had not been validated against global impact metrics. A set of prioritised metrics were therefore developed to allow the global community of innovators to assess their progress in a consistent and comparable way, and to more effectively assess the overall progress of the portfolio against the global challenge of reducing maternal and neonatal mortality.

The Saving Lives at Birth theory of change and impact framework with prioritised metrics is needed to map how the initiative contributes to the achievement of improved health outcomes for women and newborns around the time of birth, to reduce the numbers of stillbirths, and to measure progress towards this goal. This paper reports on the development of a theory of change and impact framework, in addition to the development of prioritised metrics for Saving Lives at Birth following the end of the initial commitment from the partners. The paper aims to assess the success and utility of implementing this process for such a diverse, existing programme.

## Methods

### Development of the theory of change and impact framework with prioritised metrics for existing programmes

A review of the literature regarding the creation of a theory of change and impact framework with prioritised metrics for an existing program was conducted, in addition to assessing other global health programs implemented by partners but outside the Saving Lives at Birth platform. We searched PubMed and the grey literature for references relating to the development of a retrospective theory of change and impact framework for global maternal and newborn health (MNH) programs (Additional file 1). Literature searches and scoping yielded insufficient evidence regarding the use of these tools for existing programs and nothing on retrospective design or utility. Therefore, additional inputs from technical and policy experts were sought, in conjunction with the review of existing models and reports from other organisations (such as Comic Relief and DFID) [1, 12]; these findings informed our methodology.

### Articulating the theory of change

Key programme documents were collated and reviewed in detail to enable a holistic understanding of the functionality and the organisational architecture and dynamics of Saving Lives at Birth, while recognising the different perspectives of the stakeholders (particularly partners or innovators). These documents included: each partner’s vision for programme success based on their own institutional priorities (in February 2013), the four requests for applications (RFAs), in addition to publicly available materials via the Saving Lives at Birth website [9]. A gap analysis of the Saving Lives at Birth portfolio was produced by the program to look at the domain *(science/technology, service delivery, demand creation)*; the target population *(maternal, neonate, stillbirth)*; where along the continuum of care funded innovations focussed *(such as antenatal, intrapartum, postpartum* etc.*)*; and the innovator’s country of work *(according to the World Bank country lending classification* [14]*)*. We reviewed the gap analysis to maximise our understanding of the programme and to map investments to-date.

The early scoping exercise emphasised the importance of capturing the various perspectives and roles of the different stakeholders in order to accurately identify their placement and define their interconnected pathways within the theory of change (see Table 2). In order to capture the partners’ unified objective to save lives at birth, while capturing their complimentary roles, the visions of success for each of the partners, based on their own respective organisational strategies, were reviewed. A matrix framework was created by dissecting the inputs, outputs, outcomes and impact defined by each partner. The individual frameworks were integrated to form a summary of the overall vision of success for the Saving Lives at Birth initiative. This contributed towards our understanding of roles the partners play and their placement in the program, providing an important focal-point for the articulation of the theory of change.

**Table 2:** Mapping the perspectives of actors captured in the theory of change

Perspective	What it can describe
Partner/ Stakeholder	- Demonstrates engagement of stakeholders.
	- Depicts roles and responsibilities of partners across the program.
	- Depicts how partners link with innovators perspective to provide support throughout the program.
Innovator	- Describes support available to innovators.
	- Depicts how far innovators can go with specific grant.
	- Provides insight into what is needed (outside of the program) at later stages to move towards sustainable impact.
Health policy makers, providers, and beneficiaries	- Actors external to the program that need to be engaged.
	- Describes network needed and links to innovators in order to reach sustainable impact.

Evaluation of the visions of success and strategic goals of each partner, gap analysis, and program document review were then synthesised to form the preliminary articulation of the theory of change for Saving Lives at Birth. The theory of change was further developed through an iterative process, informed by a series of consultations with the partners. A description of the processes and products with their underlying assumptions was developed for each component of the theory of change (Additional file 2).

### Review and refinement of the theory of change

Once a working model of the theory of change was articulated, innovators were encouraged to participate in a process of refinement and help further inform the theory of change from the perspective of the innovators. The theory of change was presented at the Development^X^Change in July 2014 to attending participants. The Development^X^Change is an annual Saving Lives at Birth event bringing together the partners, development experts, private companies, implementing organisations, and the community of innovators – both current grantees and finalists competing for funding. It serves as a platform for the final review of finalists for the next year’s round of funding, as well as an opportunity for all stakeholders to communicate results and lessons learned, participate in capacity-building sessions, and network to form new collaborations. The Development^X^Change is an integral part of the Saving Lives at Birth program, with the ultimate goal of continuing to catalyse accelerated impact.

The 2014 Development^X^Change was a pivotal opportunity for gaining insight directly through a wide range of Saving Lives at Birth stakeholder consultations. A series of workshops were held with partners, finalists and current innovators across all funding stages. With the theory of change and impact framework at the centre of the discussions, we held consultations with the community of innovators to gain feedback on these models, inform refinements and discuss the process for metrics prioritisation. These small group-working sessions were led by facilitators using the framework illustrated in Table 3.

**Table 3:** Thematic summary of innovator’s feedback (Development^X^Change)

Added value of partnership/ Saving Lives at Birth platform:
– Visibility
– Partnering and shared learning in community of innovators
– Integrated solutions
Maximising impact:
– Partnering and shared learning in community of innovators
– Engaging partners early
– Encourage all innovators to play active part in vision to impact at scale
– Need to understand: mechanisms/ risk to scale-up/ clear pathway to achieve impact
– Identifying priority areas
Specific comments and suggestion:
– Funding – small to take to scale, funding gap between seed and TTS grants
– Building capacity outside North America/ Europe
– Improving feedback mechanisms and support
– Competition process biases technology

### Development of the impact framework and prioritised metrics

A parallel process was conducted to identify a Saving Lives at Birth impact framework and set of prioritised metrics to measure progress towards meeting the goals of the program. This process was iterative and not in isolation of developments regarding the theory of change. The Saving Lives at Birth program represented a unique challenge because it demands flexibility within the impact framework to allow for the broad scope of differences between the types of innovation and investments. These can range from small-scale projects, such as product testing, to those of a larger-scale, such as community participation projects. The scope of these differences is not only a matter of scale, but also nature, because an innovation could be anything from a drug, to a diagnostic device, to a health insurance program. For Saving Lives at Birth, this process offered an opportunity to identify and define common elements across the program which was helpful to better understand given the varied and sometimes disparate inputs.

A comprehensive review was undertaken to identify candidate indicators for measuring progress along the impact framework. Three key types of data sources were reviewed, including: (1) relevant indicators tracked at a global level (*including the Commission on Information and Accountability, Global Reference List of 100 Core Health Indictors, Every Newborn Action Plan, Basic Emergency Obstetric Care and the World Health Organisation (WHO) Quality of Care monitoring frameworks*); (2) priority indicators identified by the Saving Lives at Birth partners; and (3) all project indicators reported by innovators during Rounds 1–3 of investments (*n* = 58 grants).

**Step 1:** All 65 indicators were compiled into a matrix ordered by key domains of a standard impact framework; process, coverage, impact. (Additional file 3) These indicators were reviewed by the Saving Lives at Birth technical working group^1^ for completeness.

**Step 2:** The indicator matrix was subsequently scored in two rounds for importance, utility and feasibility to the end user (e.g. health policy maker, provider, beneficiary) by 45 participants selecting between the following options: yes = 5, probably = 3, possibly = 1, no = 0. These participants included Saving Lives at Birth Partner’s representatives (n = 5); transition-to-scale innovators (*n* = 9); and seed innovators (*n* = 31) (Additional file 3).

The results were analysed in STATA based on ranking; key comparisons between samples were also reviewed, for example partner rankings compared with seed or transition-to-scale innovators.

**Step 3:** Priority was given to the impact and coverage indicators rated highly for importance, operationalisation and value to the end-user (policy makers, governments, service providers, health providers and women). A combined set of ten indicators was identified based on this ranking.

**Step 4:** As many of the grants are seed grants which aim to determine ‘proof of concept’, it is not possible to collect impact or coverage indicators for many of these. A list of five process indicators was therefore included within the framework, based on the highest feasibility ranking specific to seed grants.

## Results

### The Saving Lives at Birth theory of change

The theory of change captures the multifaceted nature of the Saving Lives at Birth program. It highlights the different perspectives within the program and the diverse, yet complimentary, roles of the various actors involved, and depicts the pathways to how these roles are interconnected and lead to the shared goal of reducing morbidity and mortality around the time of birth.

This theory of change articulates the interconnecting dynamics between partners and the global MNH community that is required for addressing global maternal and neonatal mortality through the program. The theory of change describes the relationship between partners and innovators and the shared accountability for the functioning of the system, as both are responsible for maximising the chance of success for innovations enrolled in the program.

### Partner’s perspective:

**Fig. 1:**
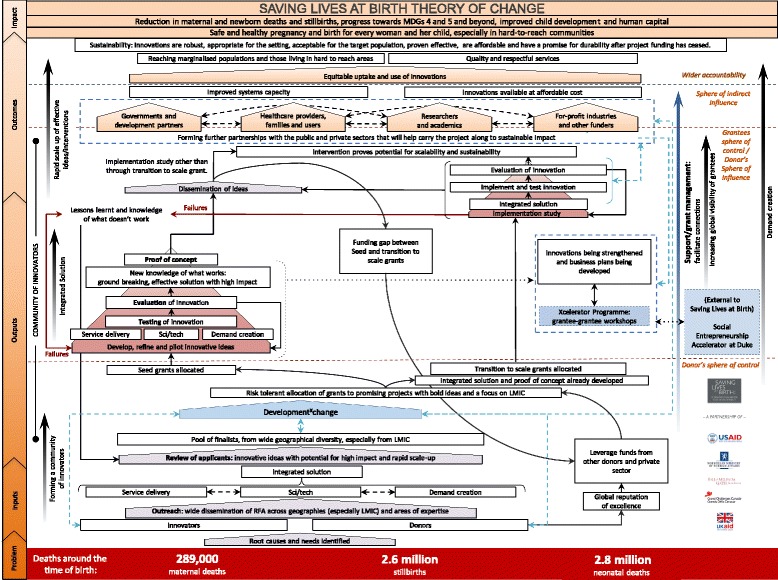
Saving lives at birth theory of change

The partner’s perspective is the first section of the theory of change and is found within the partner’s sphere of control (Fig. 1).

The partner’s perspective illustrates their responsibility for evaluating the applications for Integrated Innovation, assess the potential for transformational impact and make risk-tolerant decisions in selecting the most promising applications. A feedback loop of lessons learnt from previous rounds helps to rationalise these decisions, and provides and essential opportunity for professional reflection. Nominations for awards are made at the Development^X^Change, which links together the pool of finalists, other past and present innovators, partners, and other relevant stakeholders across the public and private sectors.

Furthermore, the theory of change highlights the shared accountability for maximising the chance of success. As represented by the blue arrow extending on the right hand side (Fig. 1), this is addressed by the partners through on-going support and award management offered throughout the program, including facilitating connections to relevant partners and/or networks who could increase an innovation’s potential for sustained impact.

### Seed Innovator’s perspective

Figure 1 also highlights the theory of change from the seed innovators’ perspective and illustrates the two-year award allocated to each seed innovator for development, piloting and, potentially, refinement of the innovation. The goal of a seed grant is to move as close as possible to proof of concept which is defined by Saving Lives at Birth as innovations which demonstrate strong evidence in a controlled or limited setting, of the achievement of promising health outcomes and/or the significant reduction of barriers to health [9]. This theory of change articulates the key concepts and stages that should be addressed by innovators throughout the development process and inbuilt evaluation cycles. It aims to reflect the integral feedback loops that are imbedded within the Saving Lives at Birth process. The program is risk-tolerant, expecting only a few awards to be highly successful and game-changing; however, it contains a strong mechanism for learning from less successful innovations in order to precipitate an improvement cycle. The programme promotes and facilitates sharing of new MNH knowledge generated through the program, including lessons learnt, to the wider global community.

Feedback from innovators who attended the Development^X^Change workshops was analysed for emerging themes and 25 key points were summarised as shown in Table 3. The most common theme identified was ‘Partnering and shared learning in community innovators’ (8 out of the 25 (32%) feedback points). The second most common theme identified were ‘Visibility’ and ‘Encourage all innovators to play active part in vision to impact at scale’ (3 (12%) of the feedback points). These findings suggest that participants strongly value the Saving Lives at Birth brand and the ability to collaborate and share learnings across a community with focus on scale from the start.

Overall, the draft theory of change was well received by the innovators participating in the workshops. However, it was strongly suggested to better portray the perceived gap between seed and transition-to-scale grants, as few innovations can move directly from a seed to a transition-to-scale grant without additional refinements and repeating the application process. This gap has been addressed for Round 5 of applications, with the inclusion of a “validation award”; however, it is not captured in the theory of change due to its retrospective nature.

The theory of change brings attention to the point along an innovation’s development process where scalability and sustainability need to be assessed. It highlights how innovators whose ideas have achieved proof of concept can return to the Saving Lives at Birth program (Fig. 1) if they wish to seek continued funding under the program to transition their innovation to scale. These ideas must “re-compete” and are reassessed for their continued potential for transformational change based on their potential for sustained impact. Innovators may also seek support for scaling up outside of the programme.

To ensure that the complex challenges of scale and sustainability are considered from the start, Saving Lives at Birth supports innovators to participate in acceleration workshops to identify and plan for the challenges they will face as they seek proof of concept and transition-to-scale by linking innovators to supportive parallel programs—specifically, the Xcelerator Program, run by Venture Well, and the Social Entrepreneurship Accelerator at Duke (SEAD). Both programs are shown in the centre of theory of change (within the innovators sphere of control and partners sphere of influence) (Fig. 1). These programs aim to facilitate innovators in the development of strategic plans for scale and sustainability, and are directly linked to creating networks for influence.

### Transition-to-scale Innovator’s perspective

There are many similarities between transition-to-scale and seed innovators regarding the pathways transitioning to impact as outlined in this theory of change (Fig. 1). Clear feedback and evaluation mechanisms are again integral to transition-to-scale grants, in conjunction with dissemination of innovative MNH ideas to the global community. However, the overall aim for a transition-to-scale grant is to prove the innovation’s potential for scalability and sustainability in order to achieve impact and save lives in LMICs. Therefore, forming MNH partnerships with governments, key stakeholders, health professionals, communities, researchers, academics, for-profit industries and other potential funders for whom a project’s scalability and sustainability is a priority is essential for these awards to succeed. This is shown within the theory of change using interconnected spaces to illustrate the cultivation of these networks.

### Underlying assumptions

When developing a theory of change it is important to consider what assumptions are being made at each step. These assumptions represent things that are not necessarily within the control of the programme but which must be in place in order for the next step in the theory of change to occur. These can then be tested to ensure they are reasonable and subsequently used to identify program barriers to achieving impact. Additional file 2 details the assumptions made at each step during this process.

### Impact at scale

As innovators move up the theory of change and achieve delivery at scale, the success of the innovation is influenced by factors outside the programme. It is then up to actors, external to the program, (e.g. governments, policy-makers, implementing organizations, funders and private-sector) to take these ideas and make informed decisions in drafting and implementing policies that are catered specifically to the context.

The ceiling of accountability is the stage where Saving Lives at Birth realises its key objectives (Fig. 1). High risk tolerance during the selection process is integral to Saving Lives at Birth, ensuring a wide range of potential innovations are funded, and hence few will successfully reach widespread scale. However, innovations that do succeed should have been found superior to alternatives in their effectiveness, affordability, robustness, appropriateness for the setting and acceptability for the target populations. Furthermore, they must demonstrate a promise for sustainability after project funding has ceased; ultimately leading to a safer pregnancy and childbirth, and a reduction in maternal and newborn deaths, and stillbirths for all. It is therefore also essential for the Saving Lives at Birth prioritised metrics toolkit to capture standardised impact measures across all funded innovations. This will ensure essential impact data are captured for those few innovations that transcend the accountability ceiling, and will ultimately inform future investment selection and development.

### The Saving Lives at Birth impact framework and prioritised metrics

**Fig. 2:**
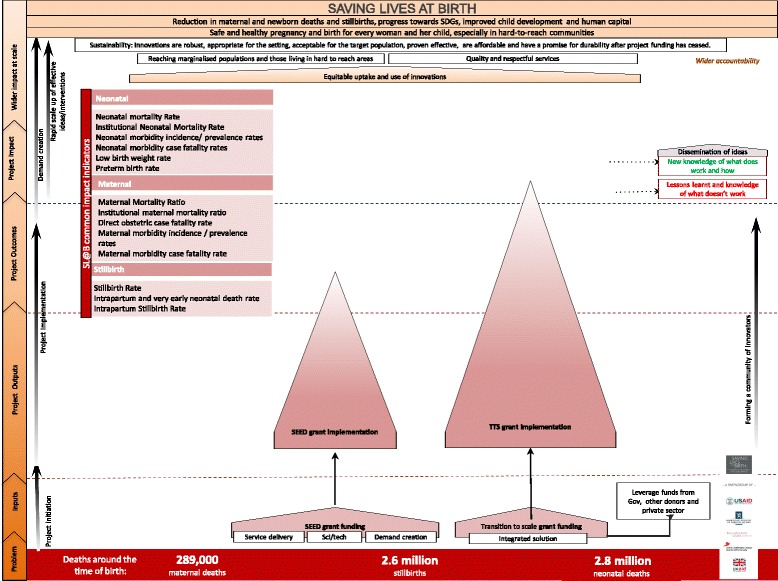
Saving Lives at Birth impact framework

The Development^X^Change workshops offered granularity to a variety of key challenges faced by innovators and demonstrated that an operational impact framework with prioritised metrics and a supporting toolkit for users were necessary. Taking into account existing monitoring frameworks for innovators, these tools were developed with the aim of facilitating implementation, tracking progress at project level and creating a set of prioritised metrics prioritised for use across all Saving Lives at Birth funded projects, and allowing comparison and data pooling between innovators. The Saving Lives at Birth retro-fit impact framework is shown in Fig. 2 and the supporting toolkit is available in Additional file 3.

### Scoring and ranking the metrics

Of the 82 participants invited to score, 45 completed the ranking exercise. Metrics were prioritised by impact and coverage indicators that rated highly for importance, operationalisation and value to the end-user. A combined set of indicators was identified based on this ranking (Table 4).

**Table 4:** Impact & Outcome Indicators by highest rank order for importance, operational and value to end user

	Ranking by All (*n* = 45)
1	Institutional Neonatal Mortality Rate
2	Intrapartum and very early neonatal death rate
3	Neonatal Mortality Rate
4	Institutional maternal mortality ratio
5	Direct obstetric case fatality rate
6	Intrapartum Stillbirth Rate
7	Maternal Mortality Ratio
8	Neonatal morbidity incidence/ prevalence rates
9	Stillbirth Rate
10	Maternal morbidity incidence/ prevalence rates

The Saving Lives at Birth prioritised metrics are illustrated in Additional file 3 and are presented according to a standard impact framework with indicators shown for impact (14 indicators), coverage (6 indicators), process (11 indicators) and outputs (11 indicators). There are 42 prioritised metrics for Saving Lives at Birth projects to report on; these include both high ranking indicators as scored by a sample of partners and innovators, including priority indicators for SL@B partner organizations to report to their stakeholders. Interestingly 26 of the final 42 prioritised metrics are also ranked in the top 10 for at least one sample (innovators, technical working group, or all).

Due to the diverse nature of the portfolio it became evident early within the indicator grading process that flexibility would be required. Because Saving Lives at Birth supports projects in various stages of development across three domains—science/ technology, service delivery and demand creation—the portfolio is extremely varied. Seed grants, for example, are aiming to reach proof of concept rather than impact at scale. These projects are therefore unlikely to be able to collect impact data to the same extent as other grants and, consequently, demand an appropriate set of metrics. The indicators for process are available in Additional file 3. Indicators marked with an asterisk(*) are the top 5 highest ranked for feasibility of measurement within seed grants. In addition, seed grants might be seeking proof of concept for a wide range of innovations as detailed above. In rounds 1–3 alone, innovations included a heat-resistant, non-injectable formulation to treat post-partum haemorrhage, low-cost technology for electricity-free oxygen concentration, and a community–led health cooperative model. These interventions all have different biological or socio-economic mechanisms and operate at different levels of the health system. Therefore, different metrics are required to capture their progress and success. Consequently, each metric will be assessed by the project for its relevance and to decide if the project is able to collect the required data.

## Discussion

Theory of change is a relatively new concept in global health, originating from the development of complex community initiatives in the United States [2]. Although it is now gaining traction, its potential has yet to be fully realised, including within the Grand Challenges platforms [1]. We sought to apply this concept to an important Grand Challenge, Saving Lives at Birth, and developed both a theory of change and an impact framework with prioritised metrics. In this work, we encountered many challenges, and gained interesting insights into the different factors involved. The theory of change maps out the paths of multiple actors (partner, innovator and end-user) which are interconnected to achieve a desired outcome [12]. This work provides an overview of the steps innovators and partners should be aiming towards, in addition to identifying what and who should be kept in mind at all stages. There is no set definition on how a theory of change should take shape and be presented. Traditionally, they are articulating during the planning phases of interventions and before implementation. It should be noted that the theory of change developed here has been structured in order to map out the paths to change on the program level, not for a single intervention, and consequently lacks granularity around specific paths to change on the level of the seed or transition-to-scale grantee perspective. However, the perspective that this theory of change takes enables monitoring of programme performance as well as project planning at a high organizational level. Further efforts can be devoted to articulating each stream within the program-level theory of change in greater detail.

The Saving Lives at Birth program is diverse with a wide range of innovations. In contrast to earlier global health grand challenges, which initially focused to address a single key challenge with biomedical innovations, this program encompasses both a broad remit, ending preventable maternal and neonatal deaths and stillbirths, and encourages innovation across all sectors from biomedical to wider determinants of health [15]. This philosophy created challenges in developing the theory of change as we needed to ensure that every link and pathway to change within the program was suitably captured and expressed. These challenges informed the construction of the impact framework, which works in support of this theory of change in order to track the program’s progress towards achieving its end-goals. However, the wide diversity across the Saving Lives at Birth portfolio is difficult to capture, even with the addition of the impact framework. Moreover, Saving Lives at Birth aims to be bold and take high risks in investments in order to yield high creativity and innovation while simultaneously striving for impact; a multifaceted approach was required to ensure that inputs into the design of the impact framework and prioritised metrics were appropriate to the spirit of the program.

Although it proved to be challenging to develop a theory of change after program activities had started, the overall process yielded some additional benefits. It required a thorough review of existing key program documents, analysis of systems and feedback from existing innovators. This process forced critical thinking and honest discussions, which have been valuable in understanding the dynamics of a complicated program, highlighting areas where the programme is excelling and where it is vulnerable. Furthermore, it has provided insight into the key areas to improve moving forward. In line with Vogel’s observations, this theory of change is more than just a tool static in time, but part of a dynamic process to support and steer critical thinking processes throughout the program cycle [1]. Other existing programs considering developing a theory of change after program inception could consider commissioning the work alongside a formal parallel program evaluation for strategic planning moving forward.

Similarly, the broad nature of the program proved challenging during the development of impact measures since they are not necessarily the same across all the types of grants and domains within the Saving Lives at Birth platform. Different indicators are required for measuring the success of an innovation across the very wide range of supported projects, covering all aspects of MNH health with a wide range of components from more general health areas. Examples of supported Saving Lives at Birth projects include those focused on nutrition, infection prevention and treatment, and water and sanitation, to specific labour and delivery care innovations, or innovations targeting the postnatal period [9]. In addition, these innovations can be located anywhere along the discovery, development, dissemination pathway, from early prototypes of a drug or a device, to innovations addressing health systems supply and demand factors, to scaling up of a complex health package.

To amalgamate this rich and diverse portfolio of innovations, a prioritised metrics list and toolkit were developed to allow for consistency and comparability across the program. The prioritised metrics list was derived by a consensus process, drawing both from metrics already in use across these programs and from other key global initiatives. The benefits of such an approach include comparability with external programs, enabling the assessment of the overall progress of the portfolio. However, within the framework for the development of these prioritised metrics, whilst good provision for coverage and impact indicators was included, standardised tools to allow comparison of prototypes were less well represented. The toolkit therefore provides a selection of prioritised metrics for innovators to utilise following assessment of their program needs, capacity and the appropriateness of the indicator to each specific project.

Although we made every effort to be inclusive in the scoring of the metrics prioritisation, the scoring of potential prioritised metrics was only undertaken by partners and innovators due to budgetary and time constraints. Despite the perspective of the end-user being a critical part of the Saving Lives at Birth Program, the current employed methodology did not allow for consideration of this important outcome. Further work is required to develop measures to assess the contribution of this to the theory of change, including the perspective of key end-users such as public health care system decision makers. Here the flexibility and evolving nature of the Saving Lives at Birth platform are a great strength as the annual Development^X^Change provides an excellent opportunity to include new learning into practise*.*

### What next

Developing the theory of change and impact framework with prioritised metrics has provided an excellent opportunity to formalise the underlying culture of programme relevant reflection while also ensuring this process is institutionalised. Furthermore, this work provides a platform for prospective theory of change as internal cycles of self-evaluation and improvement continue.

The theory of change, impact framework and metrics have been developed; however, they need to be pilot tested, validated and further iterated upon in order to better inform decisions in up-coming rounds. A second program evaluation will further inform the iteration process.

## Conclusions

The development of a theory of change and impact framework with prioritised metrics led to a greater understanding of the pathways to change within the Saving Lives at Birth Program, an existing program. This learning will be useful to the program as it expands its portfolio in future calls for proposals. The development of prioritised metrics, and encouraging the measurement and reporting of these by all innovators where relevant, will allow a greater appreciation of the impact of Saving Lives at Birth towards its overall aim to save the lives of mothers and their babies around the time of birth. This learning has the potential to be more widely applied to other Grand Challenge platforms, to enable greater understanding of the pathways to successful solutions to some of the current greatest international development problems.

### Key messages

1. Theory of change is a dynamic tool with wide ranging utility.
  This case study demonstrates that theory of change can be used successfully at different organizational levels. Here, theory of change is providing a structured mechanism for reflection and improvement at a high organizational-level aiming to support overall governance, strategy and structure. Conversely more granular detail can be included when articulating theory of change at an operational level for program implementation.
  Here the theory of change has proved to be a sensitive tool able to capture a complex and multi-faceted portfolio of work, supported by an array of different donors all with their own nuanced vision.
2. It’s not too late: retrospective articulation of theory of change can have added value
  Key developments and improvements have been made to the SLAB initiative as a consequence of the discussion and research undertaken in support of articulating the theory of change. This case study demonstrates that theory of change is not static, and when applied to multi-round ongoing programs such as SLAB, provides and essential mechanism for stimulating and steering critical review cycles.
  Retrospective articulation of theory of change was instrumental in identifying areas of strength within SLAB, and areas of vulnerability. This has wider implications for the application of retrofit theory of change and demonstrates the potential added value, and opportunity it provides to examine performance and learn from experience.
3. Consolidating an initiative-wide impact framework and prioritized metrics requires flexibility
  During this case study a multi-dimensional approach was required to ensure that inputs into the design of the impact framework and prioritized metrics were able to accommodate wide diversity across the SLAB portfolio. Global health initiatives of this sort face unique challenges given that programs might fall anywhere along the discovery, development, dissemination pathway, and measures of success vary broadly across the wide scope of potential innovations. Consequently, additional mechanisms are needed at program level to support additional project specific reporting opportunities.

## Additional files

Additional file 1: Search terms and results. (DOCX 12 kb)

Additional file 2: Prioritised Metrics Toolkit. (XLSX 27 kb)

Additional file 3: Theory of change description and assumptions. (XLSX 47 kb)

## Abbreviations

COIA: Commission on Information and Accountability
DFID/UKAID: Department for International Development
ENAP: Every newborn acton plan
LMIC: Low and middle income country
LSHTM: London School of Hygiene and Tropical Medicine
MNH: Maternal and newborn health
RFA: Request for application
SEAD: Social Entrepreneurship Accelerator at Duke
TTS: Transition-to-scale
USAID: United States Agency for International Development
WHO: World Health Organization

## Footnotes

1 Participants listed in the Acknowledgements section
