# Supplementary material for: Saving Lives at Birth; development of a retrospective theory of change, impact framework and prioritised metrics
Source: Global Health. 2018 Jan 29;14:13. doi: 10.1186/s12992-018-0327-z (PMC5789747; doi:10.1186/s12992-018-0327-z)
Supplement: Supplementary file 1 — Search terms and results. (DOCX 12 kb) [file 12992_2018_327_MOESM1_ESM.docx]

**Additional File 1, search terms and Results**

**PubMed Search**

((((health programme or health program or program or programme))) AND ((matern* or newborn* or neonat* or intrapartum))) AND theory of change

54 articles

All titles screened **(none relevant)**

(((theory of change) AND (retrofit or retro-fit or retrospective))) AND (((design or create or write or draw* or propos* or draft* or outline or make or generat* or build* or invent*)) AND theory of change)

47

All titles screened **(none relevant)**

(theory of change) AND (retrofit or retro-fit or retrospective)

129

All titles screened **(none relevant)**

**Google Scholar-** (theory of change) AND (retrofit or retro-fit or retrospective)

**28 results** All titles screened **(none relevant)**

**Cochrane- Theory of Change**

**27 results** All titles screened **(none relevant)**

**Other literature sources searched:**

New York Academy of Medicine Grey Literature Report **(none relevant)**

Open Grey **(none relevant)**

Google scholar **(none relevant)**
